# *PCSK9* Gene E670G Polymorphism and Coronary Artery Disease: An Updated Meta-Analysis of 5,484 Subjects

**DOI:** 10.3389/fcvm.2020.582865

**Published:** 2020-11-05

**Authors:** Yan-yan Li, Hui Wang, Xin-xing Yang, Hong-yu Geng, Ge Gong, Xin-zheng Lu

**Affiliations:** ^1^Clinical Research Center, First Affiliated Hospital of Nanjing Medical University, Nanjing, China; ^2^Department of Gerontology, First Affiliated Hospital of Nanjing Medical University, Nanjing, China; ^3^Department of Cardiology, First Affiliated Hospital of Nanjing Medical University, Nanjing, China; ^4^Department of Intensive Care Unit, First Affiliated Hospital of Yangzhou University, Yangzhou, China; ^5^Department of Intensive Care Unit, Baoding First Central Hospital, Baoding, China; ^6^Department of Gerontology, Nanjing General Hospital of Nanjing Military Command, Nanjing, China

**Keywords:** pcsk9, polymorphism, coronary artery disease, genetic, E670G

## Abstract

**Objective:** Research has shown a possible relationship between the E670G polymorphism of the *proprotein convertase subtilisin/kexin type 9* (*PCSK9*) gene and an increased risk of coronary artery disease (CAD). However, there is no clear consensus on the subject because of conflicting results in the literature. The current meta-analysis was performed to better elucidate the potential relationship between the *PCSK9* gene E670G polymorphism and CAD.

**Methods:** There were 5,484 subjects from 13 individual studies who were included in the current meta-analysis. The fixed- or random-effects models were used to evaluate the pooled odds ratios (ORs) and their corresponding 95% confidence intervals (CIs).

**Results:** The current meta-analysis found a significant association between *PCSK9* gene E670G polymorphism and CAD under allelic (OR = 1.79, 95% CI = 1.42–2.27, *P* = 1.00 × 10^−6^), dominant (OR = 2.16, 95% CI = 1.61–2.89, *P* = 2.22 × 10^−7^), heterozygous (OR = 2.02, 95% CI = 1.55–2.64, *P* = 2.47 × 10^−7^), and additive genetic models (OR = 1.92, 95% CI = 1.49–2.49, *P* = 6.70 × 10^−7^).

**Conclusions:** *PCSK9* gene E670G polymorphism was associated with an elevated risk of CAD, especially in the Chinese population. More specifically, carriers of the G allele carriers of the *PCSK9* gene may be predisposed to developing CAD.

## Introduction

Coronary artery disease (CAD) is an important disease threatening human health. CAD-associated morbidity and mortality have been increasing annually in China due in part to an aging population, and epidemiological studies show that dyslipidemia is an important risk factor for CAD (1). Dyslipidemia takes into account the levels of various types of lipids in the plasma and is defined either by elevated levels of triglycerides (TGs) or low-density lipoprotein (LDL-C) levels or decreased levels of high-density lipoprotein (HDL-C). Among these factors, the elevated LDL-C level has been identified as the most important risk factor in the formation of atherosclerotic plaques (2). Clinically, lowering the blood cholesterol and LDL concentration has become the dominant strategy for the cardiovascular protection in recent years.

*Proprotein convertase subtilisin/kexin type 9* (*PCSK9*) gene belongs to proprotein convertase family encoding the protein called neural apoptosis-regulated convertase-1 (NARC-l), a unique proprotein in the subtilisin family that plays an important role in the cholesterol metabolism. PCSK9 overexpression increases plasma LDL-C levels by downregulating LDL receptor (LDLR) expression after transcription. The *PCSK9* gene E670G polymorphism is associated with the coronary atherosclerosis severity and could serve as an independent predictor of the high LDL-C levels in the future.

The human *PCSK9* gene is located in lp32.3, spans 29 kb, and contains 12 exons. It encodes NARC-l with 692 amino acid residues. NARC-l is mainly expressed in liver and intestine and incises several common sequence variations of PCSK9. These variations have great effect on the plasma cholesterol level (3). PCSK9 is not only involved in lipid metabolism, but also participates in liver regeneration and neural differentiation and inhibits LDL-R expression.

The E670G polymorphism involves mutation of the adenosine (A) in the 23,968th position to a guanine (G). This results in the transformation of the glutamate (E) that is normally present in the 670th position of the 12th exon to a glycine (G). The *PCSK9* gene E670G mutation (23968A>G, rs505151) could enhance the hepatocytes' ability to degrade LDLR, leading to decreased elimination of plasma LDL and ultimately resulting in hypercholesterolemia and an increased risk of CAD.

Although significant research on the relationship between the *PCSK9* gene E670G polymorphism and CAD has been conducted, a clear consensus has yet to be reached. In 2016, He et al. found that *PCSK9* gene E670G polymorphism was associated with the CAD risk in Hainan and three provinces located in northeast China (TPNC) population. They also found that carriers of the G allele of *PCSK9* gene E670G polymorphism were more susceptible to developing CAD in both of the aforementioned regions (4). In 2011, Qiu and Wen got the similar conclusion in a Hunan Chinese population (5). However, in 2015, Yang et al. found no significant association between *PCSK9* gene E670G polymorphism and CAD in a population in the Suwan, located in southeastern China (6). Similarly, in 2009, Hsu et al. found the *PCSK9* gene E670G polymorphism to modulate plasma LDL-C levels, but not affect risk of CAD in a Taiwanese population (7).

To confirm whether the *PCSK9* gene E670G polymorphism was associated with CAD susceptibility, the current meta-analysis of 5,484 subjects from 13 separate studies was performed (Supplement Datasheet 1).

## Materials and Methods

### Publication Search and Inclusion Criteria

A primary search was conducted using the terms “*PCSK9*,” “E670G,” “23968A/G,” “rs505151,” “coronary artery disease,” and “polymorphism” on electronic databases of WanFang database, PubMed, the VIP database, EMBASE, the China National Knowledge Infrastructure, and the Web of Science. The resulting articles were published between 2007 and 2019, with the most recent update occurring on October 1, 2020.

The following inclusion criteria had to be met by the selected studies for our meta-analysis. Studies selected must (a) assess the association of CAD and *PCSK9* gene E670G polymorphism, (b) diagnose CAD as the coronary artery stenosis was no <50% in no less than one coronary artery measured either by coronary angiography or dual-source coronary computed tomography, (c) have a control group with a genotype consistent with Hardy–Weinberg equilibrium (HWE), and (d) were either cohort or case-control studies published officially.

### Data Extraction

Data from the articles that fit our inclusion criteria were extracted by three authors using a standardized protocol. Two were responsible for eliminating repetitive studies, whereas the third author served as an arbitrator to resolve any divergence between them. Studies that did not meet the inclusion criteria, were published in duplicate, or supplied insufficient data were eliminated. Similar data in multiple publications by similar author group were used for only one time in the present meta-analysis. Such items as the first author's name, publication year, region, genotyping method, matching criteria, the genotype number in the CAD and control groups, and sample size of CAD and controls are listed in Table 1. All of the subjects in the CAD groups take the lipid-lowering drugs as their serum lipid level and other hazard factors needed.

**Table 1:** Characteristics of the investigated studies of the association between the *PCSK9* gene E670G (rs505151) polymorphism and CAD.

**References**	**Year**	**Region**	**Ethnicity**	**CAD**			**Control**			**Matching criteria**	**Genotyping method**	**Sample size (CAD/control)**
				**AA**	**AG**	**GG**	**AA**	**AG**	**GG**			
He et al. (4)	2016	Hainan	Chinese	97	19	2	116	8	1	Age, sex, ethnicity, smoking, drinking	PCR-RFLP	118/125
He et al. (4)	2016	TPNC	Chinese	80	28	7	99	13	2	Age, sex, ethnicity, smoking, drinking	PCR-RFLP	115/114
Yang et al. (6)	2015	Jiangsu,Anhui	Chinese	138	22	0	168	14	0	Age, sex, ethnicity	PCR-RFLP	160/182
Qiu et al. (5)	2011	Hunan	Chinese	78	16	6	90	9	1	Age, sex, ethnicity, smoking, hypertension, T2DM, TC, TG, HDL-C, LDL-C	PCR-RFLP	100/100
Zhang et al. (8)	2014	Tianjin	Chinese	291	117	8	212	42	3	Age, sex, ethnicity, hypertension	PCR-RFLP	416/257
Salazar et al. (9)	2007	Chile	Non-Chinese	105	5	0	103	5	0	Age, sex, ethnicity	PCR-RFLP	110/108
Hsu et al. (7)	2009	Taiwan	Chinese	182	20	0	541	72	1	Ethnicity, cholesterol	PCR-RFLP	202/614
Slimani et al. (10)	2014	Tunisia	Non-Chinese	148	37	7	199	32	1	Ethnicity, smoking, cholesterol, BMI,HDL-C, LDL-C	PCR-RFLP	192/232
Meng and Liu (11)	2011	Guangdong	Chinese	146	19	0	166	14	0	Age, sex, ethnicity, TG	PCR-RFLP	165/180
Zeng et al. (12)	2011	Sichuan	Chinese	167	38	7	165	17	2	Ethnicity	PCR-RFLP	212/184
Mo et al. (13)	2015	Guangdong	Chinese	87	13	0	92	8	0	Age, sex, ethnicity, TG	DNA sequencing	100/100
Shan et al. (14)	2016	Xinjiang	Chinese	294	74	0	335	37	0	Age, sex, ethnicity, BMI	DNA sequencing	368/372
Que (15)	2017	Fujian	Chinese	149	30	5	58	2	0	Age, sex, ethnicity, BMI, smoking, hypertension, T2DM, TG	PCR-RFLP	184/60
Lin et al. (16)	2019	Fujian	Chinese	159	61	5	136	47	6	Age, TC, HDL-C, LDL-C, smoking, drinking	PCR-RFLP	225/189

PCR-RFLP method and case–control study design were adopted in the above studies.

*PCSK9, proprotein convertase subtilisin/kexin type 9; PCR–RFLP, polymerase chain reaction–restriction fragment length polymorphism. TPNC, three provinces of northeast of China*.

### Statistical Analyses

In the current meta-analysis, four genetic models as allelic (G allele distribution frequency), dominant (AG+GG vs. AA), heterozygous (AG vs. AA), and additive (G vs. A) were used. The odds ratios (ORs) and their corresponding 95% confidence intervals (CIs) were used to compare the relationship of *PCSK9* gene E670G polymorphism and CAD. The heterogeneity among the studies was calculated by using the χ^2^-based *Q* tests with statistical significance set at *P* < 0.05 (17). The fixed-effects model (the Mantel–Haenszel method) would be used when there was no heterogeneity among the included studies (18). If heterogeneity was present, the random-effects model (the DerSimonian and Laird method) would be used (19). The pooled OR was assessed by using *Z* test with statistical significance set at *P* < 0.05 level. The subgroup analysis stratified by the ethnicity was also performed.

The measurements of serum lipid levels including total cholesterol (TC), TG, LDL-C, and HDL-C data were expressed as mean ± standard deviation, and the comparison between groups was performed by *t*-test. The values of TG are analyzed as non-normal distribution. The TG parametric analysis is performed after the logarithmic conversion.

The HWE in the genotype number of control group was evaluated by using the Fisher test with significance set at *P* < 0.05 level. The potential publication bias was assessed by using the funnel plot. The funnel plot symmetry was evaluated by using Egger's linear regression test on the OR with significance set at *P* < 0.05 level under the allelic genetic model (20). The statistical analyses were performed by using Review Manager 5.3 and Stata 12.0 (StataCorp, College Station, TX, USA).

## Results

### Studies and Populations

Data were abstracted from 2,667 CAD cases and 2,817 controls (Table 1) (4–16). Of the 21 articles obtained after the initial retrieval process, 13 were eligible for the present meta-analysis. Three of the eight excluded articles were of review character, and two articles did not have a control group that followed HWE (21, 22). The remaining three excluded articles were unrelated to either the *PCSK9* gene E670G polymorphism or CAD (Supplement Datasheet 2).

### Pooled Analyses

**Figure 1:**
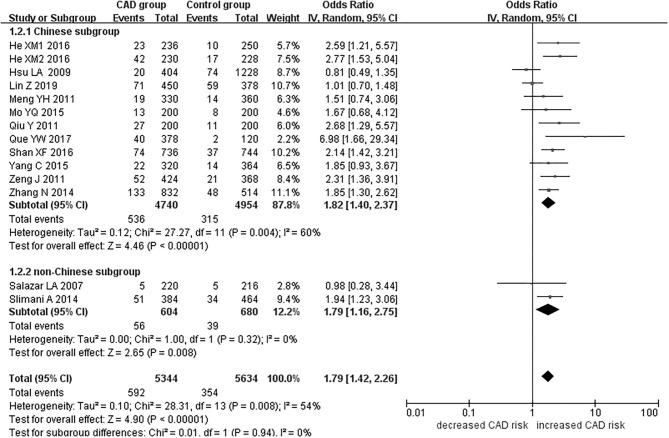
Forest plot of CAD associated with *PCSK9* gene E670G polymorphism under an allelic genetic model (distribution of G allele frequency of *PCSK9* gene E670G polymorphism).

**Figure 2:**
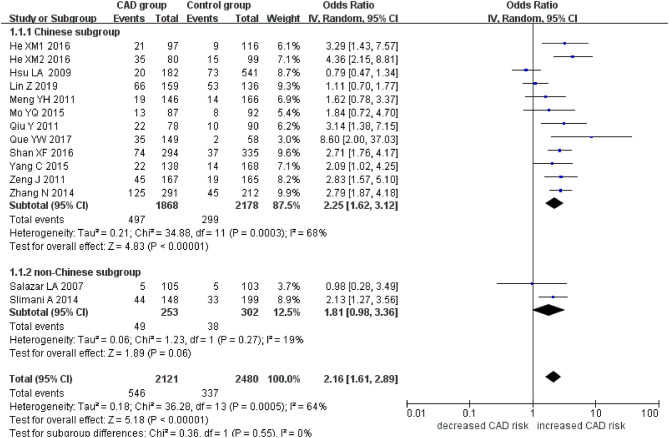
Forest plot of CAD associated with *PCSK9* gene E670G polymorphism under a dominant genetic model (AG+GG vs. AA).

**Figure 3:**
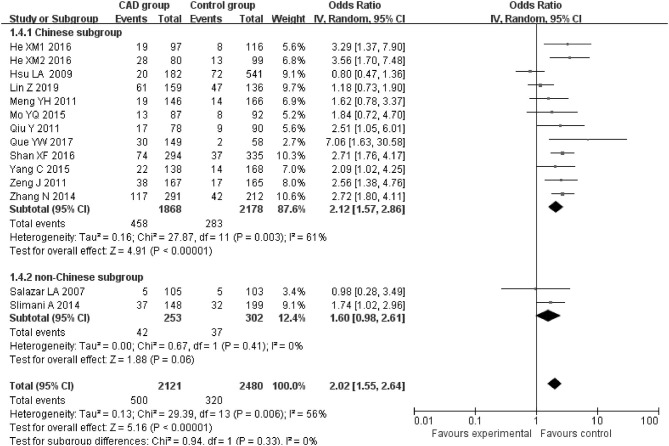
Forest plot of CAD associated with *PCSK9* gene E670G polymorphism under a heterozygous genetic model (AG vs. AA).

**Figure 4:**
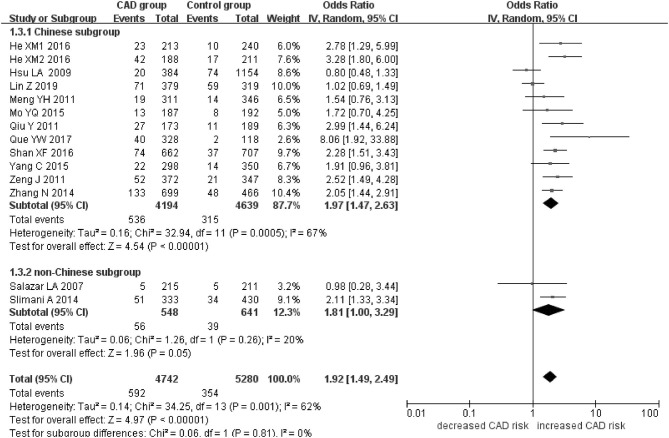
Forest plot of CAD associated with *PCSK9* gene E670G polymorphism under an additive genetic model (total G allele vs. total A).

The current meta-analysis suggests a significant association between *PCSK9* gene E670G polymorphism and CAD under allelic (OR = 1.79, 95% CI = 1.42–2.27, *P* = 1.00 × 10^−6^), dominant (OR = 2.16, 95% CI = 1.61–2.89, *P* = 2.22 × 10^−7^), heterozygous (OR = 2.02, 95% CI = 1.55–2.64, *P* = 2.47 × 10^−7^), and additive genetic models (OR = 1.92, 95% CI = 1.49–2.49, *P* = 6.70 × 10^−7^) (Table 2, Figures 1–4).

**Table 2:** Summary of meta-analysis of association between *PCSK9* gene E670G (rs505151) polymorphism and CAD.

**Genetic model**	**Pooled OR (95% CI)**	***Z* value**	***P*-value**	**Literature number**	**CAD size**	**Control size**	**$P_{\text{heterogeneity}}\left(I^{2}\%\right)$**
Allelic genetic model	1.79 (1.42–2.27)	4.89	1.00 × 10^−6^*^^	13	2,667	2,817	0.008 (54%)
Chinese subgroup	1.83 (1.40–2.38)	4.45	8.59 × 10^−6^*^^	11	2,365	2,477	0.004 (60%)
Non-Chinese subgroup	1.79 (1.17–2.74)	2.66	0.008^*^	2	302	340	0.32 (0%)
Dominant genetic model	2.16 (1.61–2.89)	5.18	2.22 × 10^−7^*^^	13	2,667	2,817	0.005^*^ (64%)
Chinese subgroup	2.25 (1.62–3.12)	4.83	1.37 × 10^−6^*^^	11	2,365	2,477	0.003^*^ (68%)
Non-Chinese subgroup	1.81 (0.98–3.36)	1.89	0.06	2	302	340	0.27 (19 %)
Heterozygous genetic model	2.02 (1.55–2.64)	5.16	2.47 × 10^−7^*^^	13	2,667	2,817	0.006^*^ (56%)
Chinese subgroup	2.12 (1.57–2.86)	4.91	9.11 × 10^−7^*^^	11	2,365	2,477	0.003^*^ (61%)
Non-Chinese subgroup	1.60 (0.98–2.61)	1.88	0.06	2	302	340	0.41 (0%)
Additive genetic model	1.92 (1.49–2.49)	4.97	6.70 × 10^−7^*^^	13	2,667	2,817	0.001^*^ (62%)
Chinese subgroup	1.97 (1.47–2.63)	4.54	5.63 × 10^−6^*^^	11	2,365	2,477	0.0005^*^ (67%)
Non-Chinese subgroup	1.81 (1.00–3.29)	1.96	0.05^*^	2	302	340	0.26 (20%)

* P ≤ 0.05.

*PCSK9, proprotein convertase subtilisin/kexin type 9; CAD, coronary artery disease; CI, confidence interval; OR, odds ratio; CAD size, the total number of CAD cases; control size, the total number of control group; allelic genetic model, G allele distribution frequency; dominant genetic model, AG+GG vs. AA; heterozygous genetic model, AG vs. AA; additive genetic model, total G allele vs. total A*.

When we analyzed subgroups by ethnicity, we found the relationships between the *PCSK9* gene E670G polymorphism and CAD were more significant in the Chinese subgroup than in the non-Chinese subgroup. In the Chinese subgroup, a significant relationship under allelic (OR = 1.83, 95% CI = 1.40–2.38, *P* = 8.59 × 10^−6^), dominant (OR = 2.25, 95% CI = 1.62–3.12, *P* = 1.37 × 10^−6^), heterozygous (OR = 2.12, 95% CI = 1.57–2.86, *P* = 9.11 × 10^−7^), and additive genetic models (OR = 1.97, 95% CI = 1.42–2.63, *P* = 5.63 × 10^−6^). In the non-Chinese subgroup, there was a significant association between them under allelic (OR = 1.79, 95% CI = 1.17–2.74, *P* = 0.008) and additive genetic models (OR = 1.81, 95% CI = 1.00–3.29, *P* = 0.05), but no significant association was found under dominant (OR = 1.81, 95% CI = 0.98–3.36, *P* = 0.06) or heterozygous genetic models (OR = 1.60, 95% CI = 0.98–2.61, *P* = 0.06).

In the whole population, significant heterogeneity was detected under allelic, dominant, heterozygous, and additive genetic models (*P* < 0.05). In our subgroup analysis, however, no significant heterogeneity was detected under these genetic models in the non-Chinese population (*P* > 0.05). It suggests that ethnicity might be the heterogeneity source.

In Table 3, on the association of lipid parameters values change and *PCSK9* gene E670G mutation in the CAD patients in the included individual studies, we could observe that compared with that in the AA genotype group, the serum TC and LDL-C levels in the GG genotype group were much higher (*P* < 0.05). Because the lipid parameters could not be found in Yang et al. (6), Salazar et al. (9), and Lin et al. (16) studies, these data were not listed in Table 3. As the sample size in the GG genotype group was relatively small in some individual studies, even 0, the lipid parameters were analyzed in the AG+GG group together. The lipid parameters differences among these genotype groups were not as distinct as those between the AA vs. GG groups (Table 3). It was suggested the *PCSK9* gene E670G mutation was positively with the elevated TC and LDL-C levels.

**Table 3:** The association of lipid parameters values change and *PCSK9* gene E670G mutation in the CAD patients in the included individual studies.

**Study name**	**TC (mmol/L)**	**TG (mmol/L)**	**HDL-C (mmol/L)**	**LDL-C (mmol/L)**
He XM				
AA (*n* = 177)	4.44 ± 0.76	1.40 ± 0.80	1.11 ± 0.38	2.84 ± 0.89
AG (*n* = 47)	4.58 ± 0.67	1.49 ± 0.89	1.14 ± 0.35	2.50 ± 0.59
GG (*n* = 9)	5.10 ± 0.77^*^	1.95 ± 0.09^*^	1.04 ± 0.24	3.30 ± 0.30^*^
Qiu Y				
AA (*n* = 78)	3.90 ± 0.59	1.44 ± 0.15	0.93 ± 0.23	2.02 ± 0.43
AG (*n* = 16)	4.12 ± 0.47	2.24 ± 0.47^*^	1.02 ± 0.24	2.37 ± 0.63
GG (*n* = 6)	4.73 ± 0.29^*^	2.26 ± 0.26^*^	1.09 ± 0.22	3.21 ± 0.86^*^
Zhang N				
AA (*n* = 291)	4.07 ± 1.16	1.82 ± 0.79	1.37 ± 0.16	2.29 ± 0.77
AG (*n* = 117)	4.45 ± 1.31	1.86 ± 1.11	1.31 ± 0.25	2.46 ± 0.71
GG (*n* = 8)	5.08 ± 1.27^*^	2.07 ± 0.78^*^	1.24 ± 0.26	3.12 ± 0.93^*^
Zeng J				
AA (*n* = 167)	3.73 ± 0.80	1.50 ± 0.59	1.26 ± 0.38	2.16 ± 0.73
AG (*n* = 38)	4.18 ± 1.34	1.78 ± 1.09	1.29 ± 0.41	2.52 ± 0.98
GG (*n* = 7)	4.61 ± 1.65^*^	2.37 ± 1.51^*^	1.12 ± 0.41	2.92 ± 1.56^*^
Mo YQ				
AA (*n* = 87)	4.48 ± 0.81	0.97 ± 0.58	1.49 ± 0.47	2.12 ± 0.72
AG+GG (*n* = 13)	4.56 ± 0.97	1.05 ± 0.89	0.97 ± 0.73^*^	3.01 ± 0.83^*^
Meng YH				
AA (*n* = 146)	4.41 ± 0.72	0.99 ± 0.62	1.48 ± 0.51	2.22 ± 0.63
AG+GG (*n* = 19)	4.63 ± 1.21	1.17 ± 0.97	0.98 ± 0.84^*^	3.02 ± 0.97^*^
Shan XF				
AA (*n* = 320)	4.56 ± 0.86	2.21 ± 1.32	1.06 ± 0.24	2.78 ± 0.69
AG+GG (*n* = 48)	4.72 ± 0.87	2.06 ± 1.01	1.08 ± 0.67	2.97 ± 0.74
Slimani A				
AA (*n* = 148)	4.88 (4.04–5.44)	1.69 ± 0.86	1.00 ± 0.36	3.10 (2.38–3.70)
AG+GG (*n* = 44)	6.78 (6.47–7.00)^*^	1.97 ± 0.96	1.03 ± 0.16	4.60 (4.00–5.04)^*^
Hsu LA				
AA (*n* = 182)	5.29 ± 1.31	2.32 ± 2.07	1.04 ± 0.27	3.25 ± 1.17
AG+GG (*n* = 20)	5.28 ± 0.74	2.48 ± 1.40	1.07 ± 0.33	3.41 ± 0.60
Que YM				
AA (*n* = 149)	4.06 ± 0.92	1.59 ± 0.79	1.09 ± 0.25	2.81 ± 0.82
AG (*n* = 30)	4.47 ± 1.07	1.45 ± 0.66	1.00 ± 0.28	3.31 ± 1.07
GG (*n* = 5)	5.43 ± 0.88^*^	2.01 ± 1.19	0.95 ± 0.14	4.16 ± 0.92^*^

* *Compared with the AA genotype group, P < 0.05*.

### Bias Diagnostics

**Figure 5:**
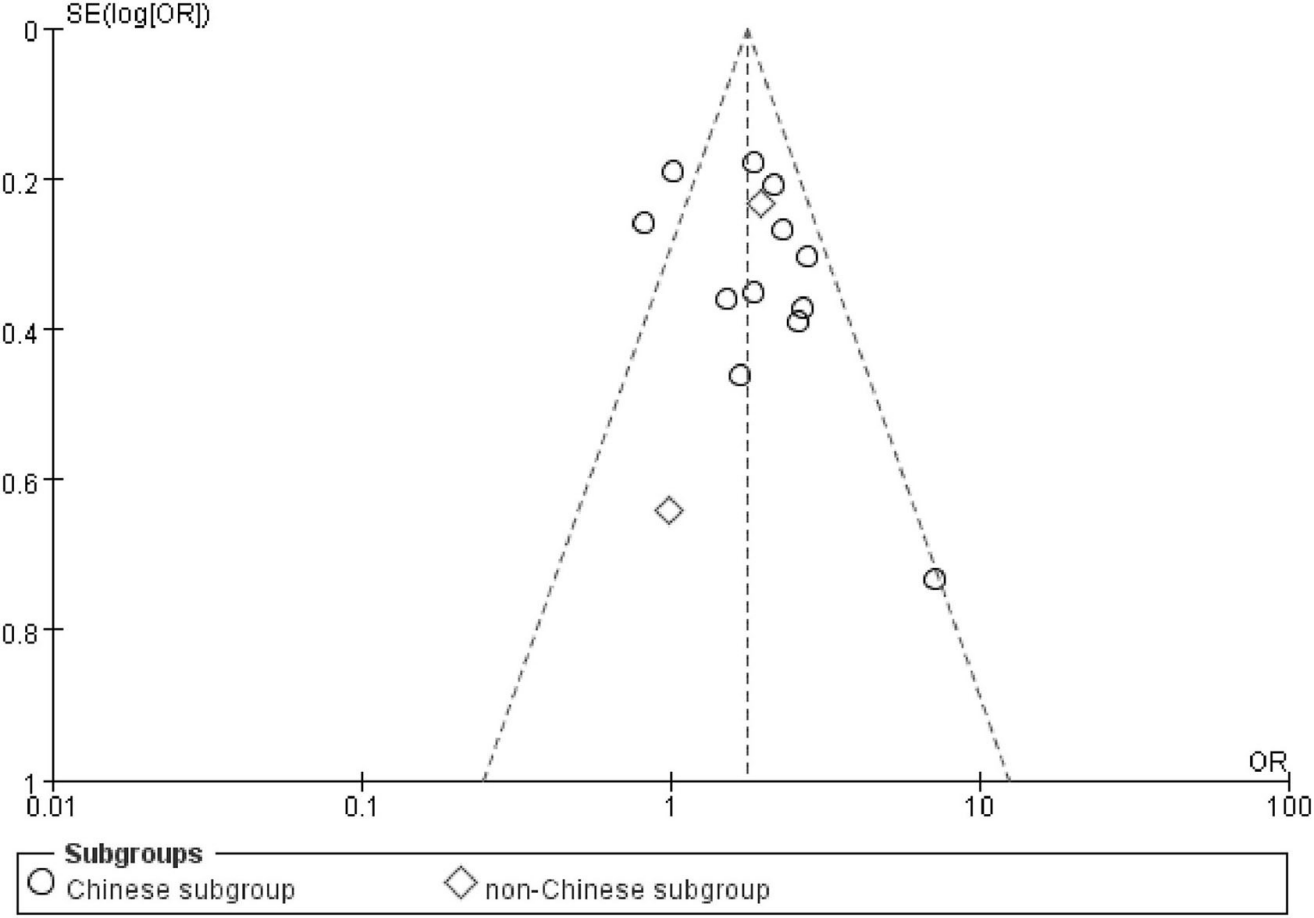
The funnel plot for studies of the association of CAD and *PCSK9* gene E670G polymorphism under an allelic genetic model (distribution of G allele frequency of *PCSK9* gene E670G polymorphism). The horizontal and vertical axis correspond to the OR and confidence limits. OR, odds ratio; SE, standard error.

**Figure 6:**
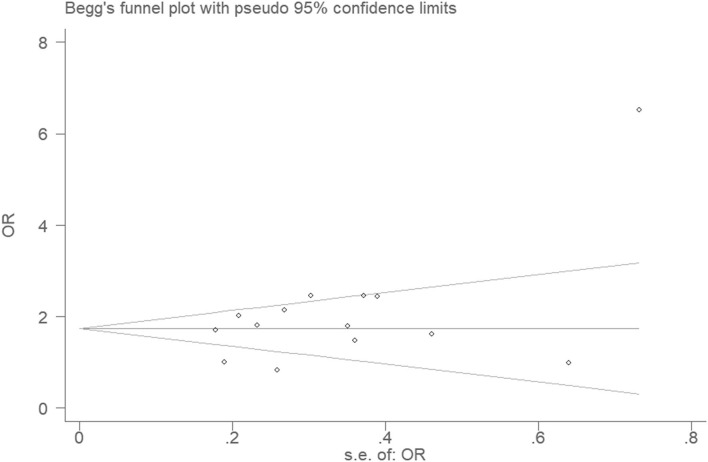
Begg's funnel plot for studies of the association of CAD and *PCSK9* gene E670G polymorphism under an allelic genetic model (distribution of G allele frequency of *PCSK9* gene E670G polymorphism). The horizontal and vertical axis correspond to the OR and confidence limits. OR, odds ratio; SE, standard error.

There was no visible publication bias in the funnel plot under the allelic genetic model (Figure 5). Furthermore, Egger's test found no significant difference indicating no publication bias in this meta-analysis under allelic genetic model (*T* = 1.87, *P* = 0.086) (Figure 6).

### Sensitivity Analysis

**Figure 7:**
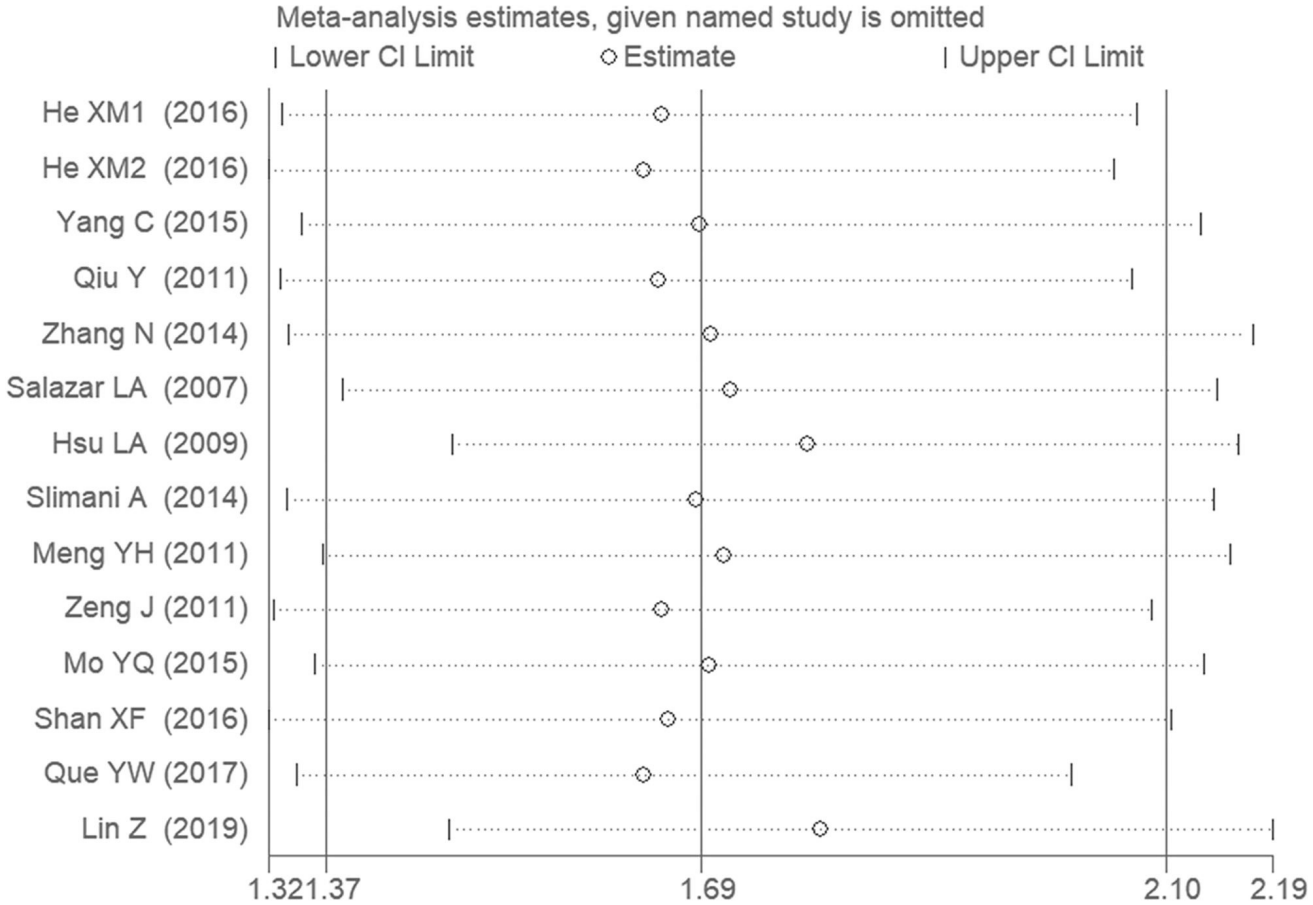
The sensitivity analysis on the association of CAD and *PCSK9* gene E670G polymorphism under an allelic genetic model (distribution of G allele frequency of *PCSK9* gene E670G polymorphism).

The sensitivity analysis was performed under the allelic genetic model. Removal of any one individual study did not affect our main result, suggesting that the results are relatively stable (Figure 7).

## Discussion

Our meta-analysis found a significant association between the *PCSK9* gene E670G polymorphism and CAD. More specifically, carriers of the G allele of the *PCSK9* gene E670G polymorphism are predisposed at an increased risk of CAD. When we stratified the data by ethnicity, we found that individuals of Chinese descent were particularly sensitized to developing CAD by the genetic mutation (*P* < 0.05). In individuals of non-Chinese descent, the significant association was only found under the allelic and additive genetic models (*P* < 0.05). However, there are only two non-Chinese studies in the current meta-analysis. The actual effect of *PCSK9* gene E670G polymorphism on CAD in the non-Chinese population is not distinct yet.

Heterogeneity was detected under allelic, dominant, heterozygous, and additive genetic models in the whole population (*P*_heterogeneity_ < 0.05), but our subgroup analysis found heterogeneity to be limited to the Chinese group (*P*_heterogeneity_ < 0.05) with no heterogeneity found in the non-Chinese subgroup (P_heterogeneity_> 0.05). This suggests that ethnicity was the primary source of heterogeneity (*P*_heterogeneity_ < 0.05).

In patients with CAD, plasma lipid levels are an important predictor of morbidity and mortality, and previous research has shown *PCSK9* gene E670G mutation to be involved in lipid metabolism through multiple mechanisms. Serum PCSK9 level is closely associated with the CAD common influence factors such as high LDL-C level (23, 24). Liu and colleagues' research verified that PCSK9 was positively correlated with LDL-C level and Gensini score (25). PCSK9 also participates in the vascular inflammatory response and promotes endothelial cell and macrophage apoptosis. Liu et al. also found PCSK9 siRNA to inhibit scavenger receptor CD36 expression and ox-LDL-C–induced, THP-1–derived macrophage, and umbilical vein endothelial cells apoptosis (26).

The *PCSK9* gene E670G mutation is a gain-of-function mutation that enhances the protein's capacity to degrade the LDLR. It might be associated with Ser-phosphorylation of PCSK9, which maximizes both its secretion and activity on the LDLR (27). Not only has this been shown to elevate the plasma LDL-C level, but is also associated with autosomal dominant hypercholesterolemia or familial hypercholesterolemia (FH) (28). Hypercholesterolemia is the sole risk factor for the experimental atherosclerosis animal model, which could be replicated consistently. At normal levels, plasma lipids that enter the vascular wall can be eliminated immediately by LDLR-expressing macrophages. As plasma lipid levels increase, however, the macrophage LDLR elimination pathway is negatively regulated, resulting in the deposition and oxidation of lipids in the vascular wall. The oxidized lipids then activate the scavenger receptor pathway of the macrophage. Because this pathway lacks any sort of negative feedback, macrophages continue to take up the oxidized lipids, turn into the foam cells, and ultimately form atherosclerotic plaques (29, 30).

In the clinic, evolocumab, a fully humanized monoclonal antibody that inhibits PCSK9, is used to treat hyperlipidemia. Patients with the wild-type EE genotype patients may be more sensitive to evolocumab therapy than patients with either the EG or GG mutation. Clinicians may therefore administer a greater dose of evolocumab for patients with the *PCSK9* gene E670G mutation to achieve equivalent clinical results. That is to say, evolocumab is more suitable to reduce the serum lipid level for the EE genotype patients than EG or GG genotype patients.

Previous meta-analyses on the subject have been performed before. In 2015, Adi et al. found that *PCSK9* gene E670G polymorphism might be involved in CAD pathogenesis (31).Their study concluded that 670G carriers may be predisposed to developing CAD. Although their conclusion was similar to that of the current meta-analysis, only five eligible studies were included in their study, limiting the credibility of the study. Also in 2015, Cai et al. found a positive association between *PCSK9* gene E670G polymorphism and risk of CAD and found that the G allele carriers had higher risk of CAD than non-carriers under dominant genetic model, as well as under allelic genetic model (*P* < 0.001) (32). Because only seven eligible studies in HWE were involved and only two genetic models were applied in their study, we believe that our study may offer a more objective result. In 2019, Lin et al. got a similar conclusion on the association between *PCSK9* gene E670G polymorphism and CAD by a meta-analysis (16). In their study, they did not exclude the literatures violating the HWE (21, 22). Moreover, more studies are included in the current meta-analysis than that in Lin and colleagues'. Hence, the current meta-analysis enhanced the credibility of the results.

However, some limitations still existed in the current meta-analysis. This study does not replace the need for the multiple large-scale studies necessary to understand the precise relationship between the *PCSK9* gene E670G polymorphism and CAD. Furthermore, environmental factors such as diet, smoking, diabetes, and metabolism diseases also play significant roles in CAD development. The microeffects of many other genes relating to CAD development have yet to be fully understood (i.e., *matrix metalloproteinase-9* gene-1562C>T polymorphism, *TGF-*β*1* gene−509C/T Polymorphism) (33, 34). *PCSK9* gene D129G, S127R, R496W, and R218S polymorphisms also influence the PCSK9 activity and CAD susceptibility (35).

FH is a severe autosomal monogene dominant disease characterized by a significant increase in plasma cholesterol, xanthoxam of the skin, and early-onset coronary heart disease (36). The main pathological basis of FH is the mutation of *LDLR* gene. *LDLR* mutations occurred in 70% of FH patients (37). *Apo B* mutations have been detected in about 2–5% of cases in northern Europe, but not in other populations generally. *PCSK9* mutations that lead to the acquisition of active function only account for <5% of FH cases (38). In Table 3, it is found that the LDL-C level is much higher in the GG group than that in the AA group. The effect on CAD via this variant could be explained by the increased LDL-C. E670G mutation should be one of the gain-of-function mutations. But it is not equal to FH-causing mutation. Weak gain-of-function mutations in *PCSK9* gene may be a part of genetic background of polygenetic FH, but it is difficult to say this is the disease-causing mutation in monogenic FH with the data from the current study in Table 3 and the study of Shek et al. (39). Usually, mean LDL-C levels are >4.5 and 6 mmol/L in average in heterozygous FH and 13 mmol/L in homozygous FH. Hence, it is implied that this *PCSK9* gene E670G mutation is not pathogenic as a FH-variant according to the Dutch Lipid Clinic Network Criteria to diagnose FH in the current study (40). It is difficult to deduce that *PCSK9* gene E670G mutation is an FH-causing mutation. However, other *PCSK9* gene polymorphisms than E670G mutation are likely pathogenic variant. In 2003, Abifadel et al. found that *PCSK9*, S127R, and F216L mutations cause autosomal dominant hypercholesterolemia in French (3). In 2007, Lin et al. found that *PCSK9* gene R306S variant was pathogenic in a 14-year-old Chinese girl with FH (41). In 2019, Hori et al. reported that *PCSK9* Glu32Lys, Asp129Asn, Arg215His, and Arg496Trp mutants were regarded as pathogenic or likely pathogenic variants of Japanese heterozygous FH (42).

There is likely difference between the effect on LDL-C between Chinese and non-Chinese population in the Table 3 data. The difference of effect on CAD should be explained by the difference of effect on LDL-C. However, only two non-Chinese studies are included in the current meta-analysis. The actual effects on CAD and LDL-C in the non-Chinese population need to be verified by more studies in the future.

In brief, *PCSK9* gene E670G polymorphism was significantly associated with CAD risk, especially in the Chinese population. Persons with the G allele of *PCSK9* gene E670G polymorphism may be at an increased risk of developing CAD. More studies on the relationship between *PCSK9* gene E670G polymorphism and CAD should be performed to further verify this conclusion.

## Data Availability Statement

The raw data supporting the conclusions of this article will be made available by the authors, without undue reservation.

## Author Contributions

Y-yL and HW researched data. Y-yL wrote manuscript and researched data. Y-yL, H-yG, and X-xY reviewed/edited manuscript. X-zL and Y-yL contributed to discussion, reviewed and edited manuscript. Y-yL and GG researched data, contributed discussion. All authors contributed to the article and approved the submitted version.

## Conflict of Interest

The authors declare that the research was conducted in the absence of any commercial or financial relationships that could be construed as a potential conflict of interest.

## Supplementary Material

Supplement Datasheet 1 PRISMA checklist.

Supplement Datasheet 2 PRISMA Flow Diagram.
