# Supplementary material for: PCSK9 Gene E670G Polymorphism and Coronary Artery Disease: An Updated Meta-Analysis of 5,484 Subjects
Source: Front Cardiovasc Med. 2020 Nov 5;7:582865. doi: 10.3389/fcvm.2020.582865 (PMC7683799; doi:10.3389/fcvm.2020.582865)
Supplement: Supplement Datasheet 2 — PRISMA Flow Diagram. [file Data_Sheet_2.docx]

**PRISMA 2009 Flow Diagram**


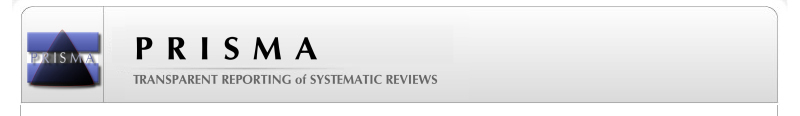


Records excluded for lacking control group (n = 0)

Studies included in qualitative synthesis
(n =13)

Records excluded for repeated publication
(n = 0 )

Records excluded for no association with *PCSK9 gene E670G polymorphism or CAD*

(n =3)

Full-text articles excluded for deviation from HWE (n =2 )

Records excluded for review characteristic
(n =3 )

Articles assessed for eligibility
 (n =13)

Full-text articles assessed for eligibility
(n =16)

Records screened
(n =18 )

Records after duplicates removed
(n =21)

Additional records identified through other sources
(n =0 )

## Identification

## Eligibility

## Included

## Screening

Records identified through database searching
(n =21 )
